# Mechanisms Mediating Pediatric Severe Asthma and Potential Novel Therapies

**DOI:** 10.3389/fped.2017.00154

**Published:** 2017-07-05

**Authors:** Aldara Martin Alonso, Sejal Saglani

**Affiliations:** ^1^Inflammation, Repair and Development Section, National Heart and Lung Institute, Imperial College London, London, United Kingdom; ^2^Respiratory Pediatrics, The Royal Brompton Hospital, London, United Kingdom

**Keywords:** severe therapy-resistant asthma, mechanisms, eosinophils, innate cytokines, therapies, remodeling, children

## Abstract

Although a rare disease, severe therapy-resistant asthma in children is a cause of significant morbidity and results in utilization of approximately 50% of health-care resources for asthma. Improving control for children with severe asthma is, therefore, an urgent unmet clinical need. As a group, children with severe asthma have severe and multiple allergies, steroid resistant airway eosinophilia, and significant structural changes of the airway wall (airway remodeling). Omalizumab is currently the only add-on therapy that is licensed for use in children with severe asthma. However, limitations of its use include ineligibility for approximately one-third of patients because of serum IgE levels outside the recommended range and lack of clinical efficacy in a further one-third. Pediatric severe asthma is thus markedly heterogeneous, but our current understanding of the different mechanisms underpinning various phenotypes is very limited. We know that there are distinctions between the factors that drive pediatric and adult disease since pediatric disease develops in the context of a maturing immune system and during lung growth and development. This review summarizes the current data that give insight into the pathophysiology of pediatric severe asthma and will highlight potential targets for novel therapies. It is apparent that in order to identify novel treatments for pediatric severe asthma, the challenge of undertaking mechanistic studies using age appropriate experimental models and airway samples from children needs to be accepted to allow a targeted approach of personalized medicine to be achieved.

## Introduction

**Figure 1:**
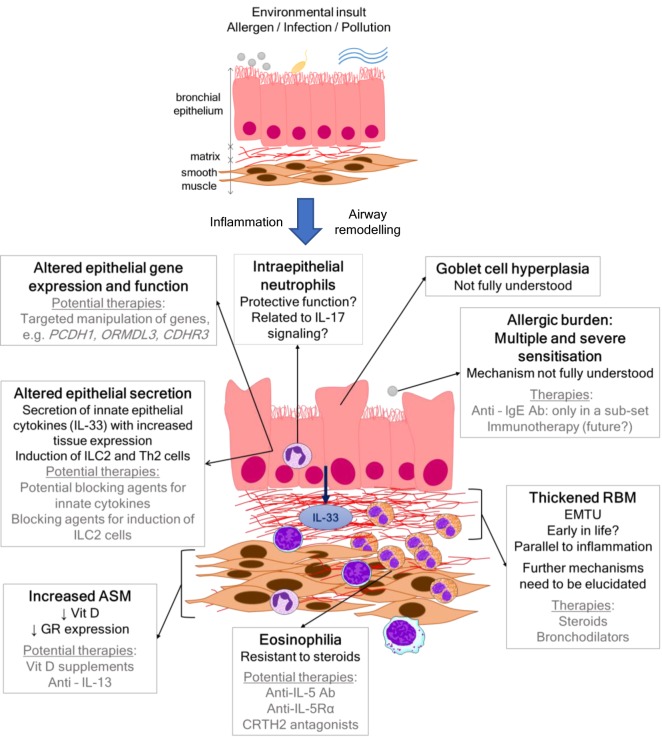
Scheme showing airway wall features, mechanisms, and current and/or potential therapies in pediatric severe asthma.

Severe asthma is thought to be rare in children, affecting approximately 2–5% (1) of all patients; however, we have little idea of the actual size of the problem (2). The proportion of health-care resources utilized by patients with severe disease is disproportionate to prevalence, whereby, they use up to 50% of all health-care costs for asthma (2). Improving control for patients with severe asthma is, therefore, a significant unmet clinical need. Pediatric asthma is a heterogeneous disease, and within that, severe asthma is also recognized to be heterogeneous with numerous clinical, pathological, and physiological phenotypes (3). It is apparent that, in order to identify novel treatments for pediatric severe asthma, the mechanisms that mediate the disease in children need to be investigated so that a targeted approach of personalized medicine can be achieved. However, mechanistic data in childhood studies are rare, partly because obtaining airway samples from children is a challenge and also because there is a reluctance to generate age-specific experimental models. This review will summarize the current data that give insight into the pathophysiology of pediatric severe asthma and will highlight potential targets for novel therapies (Figure 1). Avenues for future research and approaches that will enable mechanistic studies to be undertaken more readily in children will also be discussed.

## Diagnosis of Severe Asthma in Children

In order to accurately identify the mechanisms mediating severe pediatric asthma, it is essential that the diagnosis is correct. Objective measures supporting the key pathophysiological features of asthma including reversible airflow obstruction, airway hyperresponsiveness, chronic airway inflammation, and the presence of confirmed wheeze and breathlessness are essential in confirming the diagnosis. The ERS/ATS guidelines for the diagnosis and management of severe asthma include children (4) and must be adhered to in the assessment of these patients. In particular, the guidelines stipulate it is important that children on maximal maintenance treatment and poor control are not automatically labeled as having severe asthma. The umbrella term used to describe children who have poor control despite maximal prescribed treatment [GINA steps 4/5, or maintenance inhaled steroids ≥800 mcg daily budesonide and long-acting beta-agonists (LABAs)] is problematic severe asthma (5). Within this is a subgroup with difficult asthma, in whom, underlying modifiable factors such as poor adherence to treatment, explain persistent symptoms and poor control (6). After modifiable factors have been optimized and addressed (7), there remains a group of children with good adherence and persistent poor control, these are patients with true severe asthma (5), and will form the focus of the data discussed in this review.

## Pathology of Severe Asthma in Children: Inflammatory Phenotypes

### Airway Eosinophilia: Utility As a Therapeutic Target?

Studies that have included children with true severe asthma have shown that the airway pathology is characterized by luminal [bronchoalveolar lavage (BAL)] and tissue (endobronchial biopsy) eosinophilia (8). This eosinophilic airway inflammation persists despite systemic steroids in the majority of patients (9). Whether there is an association between airway eosinophil number and atopy (8) or not (10) is still uncertain. Although there is no disputing the steroid resistant eosinophilia that characterizes pediatric severe asthma (8), we have little idea of the functional impact of eosinophilia on disease manifestation. There is little clinical correlation between airway eosinophilia and symptom control or lung function. Murine experimental studies have shown that eosinophil-deficient mice have a similar phenotype to wild-type mice and, therefore, eosinophils play little role in the development of house dust mite (HDM)-induced allergic immunity (11) or airway hyperresponsiveness (12). It, therefore, appears that the eosinophilia does not contribute to altered lung function or daily symptom control. However, targeting eosinophilic inflammation using a monoclonal antibody to interleukin (IL)-5 has shown a significant reduction in exacerbations in adults with severe asthma (13). But interestingly, the subgroup that benefited most had both eosinophilic disease and frequent exacerbations (14). This suggests a specific impact of eosinophils in promoting exacerbations, but has not been confirmed mechanistically. Few studies that have targeted airway eosinophils have been undertaken in children with severe asthma. Disappointingly, one pediatric study that compared the effect of titrating maintenance inhaled steroid therapy according to sputum eosinophils or to clinical guidelines and symptoms-based management showed no benefit of the eosinophil-guided strategy in reducing exacerbations (15). This was in contrast to a prior study undertaken in adults (16). A possible explanation for the lack of effect in children may be that there is marked within patient variability in airway eosinophils over time which is independent of clinical disease status or changes in treatment (17). A systematic review of studies in children, which have used exhaled nitric oxide as a non-invasive surrogate marker for eosinophilic inflammation to guide treatment have shown some benefit in reducing exacerbations, but no effect on daily symptom control or maintenance treatment (18). The current evidence suggests that targeting airway eosinophils is most likely to be successful in children with a frequently exacerbating phenotype and may be less effective in those with persistent symptoms, and there is unlikely to be any benefit on improving lung function. To date, trials of the efficacy of monoclonal antibodies that target either IL-5 or its receptor have not been undertaken in children with severe asthma, but given the prevalence of airway eosinophilia in children, this seems an obvious avenue to pursue. Mepolizumab is an anti-IL-5 humanized monoclonal antibody that reduces circulating eosinophils. In adults and adolescents (aged 12–17 years), exacerbations decreased without improvement in FEV_1_ nor quality of life with Mepolizumab (13). However, only a very small number of adolescents were included. Studies specifically assessing efficacy in children with severe asthma have not yet been undertaken. However, no differences in adverse effects were observed in the adolescent group enrolled in the phase 3 trial compared to the overall population (13, 19). An ongoing clinical trial (20) studying the pharmacological properties of subcutaneous administration of Mepolizumab in children aged 6–11 years with severe eosinophilic asthma will help to determine safety in younger children. Recently, another anti-IL-5 humanized monoclonal antibody, reslizumab, has been approved by the European Medicines Agency as add-on therapy in adults with uncontrolled severe eosinophilic asthma (blood eosinophil count ≥400 cells/μl). It decreased exacerbations, improved lung function and quality life (21). It will be assessed in patients aged 12 years and older with severe eosinophilic asthma (22). Benralizumab targets the receptor for IL-5 (IL-5Rα) and in a phase III study in patients aged 12 years and above with severe uncontrolled asthma, it was well tolerated and depleted blood eosinophils, reduced exacerbation rates, and improved lung function (23). However, none of the trials that have been undertaken to assess the efficacy of blocking IL-5 in severe asthma have assessed efficacy in children alone. Therefore, at present, we do not know whether the data from the studies in adults can be extrapolated to pediatric severe disease. This is an obvious gap in our knowledge that needs to be addressed especially because of the marked pulmonary eosinophilia that characterizes pediatric severe asthma.

An important point to consider when defining biomarkers that may help to identify patients most likely to benefit from treatments that target eosinophilic inflammation is the relationship between peripheral blood eosinophils and airway eosinophils. When adherence to maintenance high-dose inhaled steroids has been optimized, and those with true severe asthma have been identified, there may be little relationship between compartments, whereby, elevated airway eosinophils may persist despite a normal blood eosinophil count (24). In adult studies, a cut-off of 0.3 × 10^9^ cell/l (25) for blood eosinophils gave approximately 75% sensitivity and specificity for sputum eosinophilia. Therefore, there is no single peripheral or non-invasive biomarker that can be used to represent airway eosinophilia, and a composite measure is likely to be most helpful.

## Dendritic Cells (DCs)

The antigen-presenting cells of the lung, DCs, capture allergens reaching the airway epithelium, process them into peptides, and load them onto the major histocompatibility complexes class II. In contrast to gut and skin, airways are immunologically immature at the time of birth (26), and DCs are not present in the airways at birth but stimuli such as microbes or pollutants can activate pattern-recognition receptors (PRRs) on epithelial cells that produce cytokines and chemokines attracting immature pre-DCs (27, 28). Activation of epithelial PRRs also results in release of cytokines, such as IL-25 (29), IL-33 (30), or thymic stromal lymphopoietin (TSLP) (31), and danger signals, such as uric acid (32), which further activate DCs. Activated DCs migrate to draining lymph nodes where along with costimulatory molecules will bind and activate T cell receptors (TCRs) on the surface of naïve CD4 T cells (33).

Two subpopulations of DCs have been identified: myeloid or conventional DCs (cDCs) or DC1 (CD11c+ CD123dim+ in cytometric analysis) and lymphoid or plasmacytoid DCs (pDCs) or DC2 (CD11c− CD123high+) (34). Apart from the cDCs, mouse studies have reported that a different subset of DCs, monocyte-derived DCs, orchestrate the pro-inflammatory environment in the airways by secreting chemokines that attract inflammatory cells during allergen challenge (35). Both mDC and pDCs take up inhaled allergen and present it to T cells in animal models of allergic airways disease (36). Depletion of pDCs during allergen challenge resulted in allergic airways disease whereas adoptive transfer of pDCs before sensitization prevented disease, suggesting a protective role of pDCs that could be applied in clinic (36).

Activated DCs can form tight junctions with the airway epithelium and detect inhaled allergens without disturbing the epithelial barrier (37) and have upregulated chemokine receptors and costimulatory molecules so they have more capacity to migrate to the lymph nodes and stimulate naïve T cells (38). In a study including 50 atopic children and 40 healthy controls, serum OX40L levels were higher in children experiencing acute severe asthma exacerbations and during stable severe persistent asthma compared to mild/moderate exacerbations and mild or moderate persistent asthma, respectively, and this correlated positively with blood eosinophil counts (39). It has been reported that sputum from asthmatic children treated with inhaled steroids contain increased airway DCs with reduced expression of the costimulator CD86, suggesting that either asthma or steroid therapy may impair DC trafficking and/or maturation reducing the pro-inflammatory responses (40). Flow cytometry analysis of DCs in cord blood of neonates from allergic and non-allergic parents and in peripheral blood of allergic and healthy children has allowed the identification of a new DC population CD11c− CD123dim+ named “less differentiated” DCs (ldDCs). This population was the predominant DC population in cord blood and decreased with age. It was also increased in children with atopic dermatitis whereas was decreased in asthmatics receiving high-dose inhaled corticosteroids. So it was proposed that ldDC could be involved in the severity of allergy/asthma. No differences in DC populations were found in cord blood from neonates with low versus high risk for allergic disorders (41). In contrast, blood pDCs were increased in both atopic and non-atopic asthmatic adults (42).

Although targeting DCs may be an attractive approach in the treatment of asthma, there are currently no specific therapies, either from experimental studies, or being tested in clinical trials that target DC numbers or function. It is still necessary to understand the complex cellular and molecular pathways involved in altering pDC function in pediatric asthma before therapeutic applications can be considered.

## Airway Epithelium: Interactions Between Innate and Adaptive Immunity in Pediatric Severe Asthma

The airway epithelium is the first site of contact between the host and environment. Allergens, viruses, and other environmental exposures directly stimulate and interact with the epithelium. It has a role not only as a physical barrier but also contributes to the development of the immune response and maintenance of inflammation. Therefore, it is not surprising that the airway epithelial barrier is altered in asthma (33, 43). In recent years, genetic studies of bronchial epithelial cells have discovered several genes, such as protocadherin 1 (*PCDH1)* (44), *periostin (POSTN), serpin family B member 2 (SERPINB2)*, and *chloride channel accessory 1 (CLCA1)* (45), associated with asthma phenotypes. Of these, *PCDH1* was very specifically associated with childhood asthma (46). Consequently, it has been suggested that many of the different pathological mechanisms underlying asthma phenotypes may originate in the airway epithelium (47).

Allergens, microorganisms, and allergen-derived protease activities not only activate DCs but also airway epithelial cells through the activation of toll-like receptors, which leads to secretion of cytokines and danger signals. These signals can be propagated through the dysregulation of the epithelial–mesenchymal trophic unit (EMTU), which is the bidirectional interaction between epithelium and mesenchyme involving the release of growth factors and cytokines, resulting in the amplification of inflammation and structural changes (remodeling) (43, 48). It is thought that the drivers of remodeling may be recruited CD34+ fibrocytes located at areas of collagen deposition and in BAL acting as myofibroblasts (49, 50), but it has also been proposed that they could stimulate the differentiation of resident mesenchymal cells (50). Epithelial cells can also transdifferentiate into fibroblasts/myofibroblasts by epithelial–mesenchymal transition (51), but this has not been proven in asthma (52).

## Innate Epithelial Cytokines and Type 2 Lymphoid Cells in Pediatric Severe Asthma

Innate immunity is being increasingly recognized as being an equal contributor to asthma pathogenesis as adaptive immunity. Upon exposure to environmental stimuli (allergens, infection, and pollution), the activated epithelium releases cytokines, such as IL-25, IL-33, or TSLP and danger signals, such as uric acid, which contribute to the onset of innate immune mechanisms resulting in disease initiation and propagation. DCs (as discussed above), mast cells (MCs) (53), type 2 innate lymphoid cells (ILC2) (54), and basophils (55) are all induced by the release of the innate epithelial cytokines. Consequently, targeting IL-25, IL-33, and TSLP is an interesting therapeutic approach for severe asthma and is actively being pursued.

Specifically, in pediatric severe asthma, we have shown increased expression of the innate epithelial cytokine IL-33 in the bronchial tissue and an association with increased levels and both airway remodeling and steroid resistance (56). More recently, we have shown that a specific sub-phenotype of patients with severe asthma and fungal sensitization have even higher levels of IL-33 in both BAL and biopsy (57). It is now also apparent that the downstream effector cells that are induced by IL-33, ILC2 cells are increased in the airways of children with severe asthma compared to non-asthmatic controls (58). Interestingly, a specific association between type 2 ILCs and severe asthma has also been demonstrated in adults (59). The ILCs present in BAL from pediatric patients were characterized by lineage negative markers (absence of the T cell antigens) and presence of the type 2 receptor CRTH2. In contrast to Th2 cells, they were a rare cell population, making up only 0.2% of lymphoid cells. Of note, however, both cell types did express CRTH2. Although increased numbers of both ILC2s and Th2 cells have been demonstrated in pediatric severe asthma, their functional and clinical relevance remains unknown, since there were no clear correlations between cell numbers in BAL and symptoms or lung function (58).

Asthma has been typically considered a Th2 disorder since the predominant inflammatory phenotype is eosinophilic as observed in BAL and endobronchial biopsies from children with severe disease (8). Although studies have investigated T lymphocytes in peripheral blood from children, data relating to the airway inflammatory phenotype have been lacking. We have recently shown that children with severe asthma have increased numbers of CD4+ T cells in BAL compared to non-asthmatic controls, and that these cells make up approximately 40% of all airway CD3+ lymphocytes and express the CRTH2 receptor (58). Interestingly, when we had previously quantified CD4+ cells in endobronchial biopsy, numbers were not significantly different to non-asthmatic controls (8), suggesting that there may be differences in the luminal and tissue compartments.

Mechanistically, naïve T cells in draining lymph nodes differentiate to Th2 cells *via* IL-4-mediated activation of STAT6 and GATA3. Th2 cells migrate to the airway mucosa and secrete the Th2 cytokines IL4, IL-5, and IL-13. But, detection of Th2 cytokines in severe asthmatic children remains controversial (3, 8). It has been generally accepted that IL-5 mediates the recruitment of eosinophils by the expression of epithelium-derived chemokines named eotaxins [CC-chemokine ligand 11, CCL24, and CCL26]. It also promotes bone marrow development and mobilization of eosinophil precursors. Children with STRA have airway remodeling and eosinophilic inflammation, but in the absence of detectable levels of Th2 cytokines, without neutrophilia nor MC infiltration (8).

## Mast Cells

Cross-linking of FcεRI following MC exposure to allergen can result in MC activation, which is characterized by degranulation and production/secretion of preformed histamine, lipid mediators, enzymes (proteases, hydrolases, cathepsin G, and carboxypeptidase), and cytokines (including tumor necrosis factor, IL-4, IL-5, IL-6, IL-13, 3 CCL3, IL-33, and granulocyte-macrophage colony-stimulating factor) (60). The arachidonic acid-derived mediators are prostaglandin (PG)D_2_, leukotriene (LT) C_4_, and platelet-activating factor, which can induce bronchoconstriction, mucus secretion, and edema (61). The wide repertoire of cytokines has several effects ranging from IgE synthesis to neutrophil and eosinophil activation to fibroblast growth. Similarly, the secreted enzymes have wide effects, such as degradation of allergens, enhanced airway smooth muscle (ASM) contractility, and enhanced IL-33 activity (60). But the role of MCs in asthma is not based solely on its products but also on their strategic location. Whereas in healthy airways MCs are predominantly located near blood vessels and within the lamina propria (62), MCs tend to relocate to the airway epithelium (63), submucosa (64), submucosal glands (65), and ASM (66) in asthma. However, these data are all from adult studies. The contribution of MCs to pediatric asthma is less well known. Several studies have reported that MC frequency was similar in the subepithelium (8, 10, 67, 68) and in the ASM (8, 69) between wheezing or asthmatic children and controls. In a recent study in biopsies of severe asthmatic children, it was reported that ASM MC numbers were associated with the number of severe exacerbations and eosinophilia, but not with remodeling or lung function (70).

Despite the apparent importance of MCs in the pathology of severe asthma, to date, therapies that are MC stabilizers such as cromolyn sodium and nedocromil that inhibit MC degranulation have proven very disappointing in the clinic. MC predominance in the airway submucosa and epithelium has been associated with severe asthma in adults because of increases in PGD_2_ levels, which is produced mainly by MCs but also Th2 cells, macrophages, and eosinophils (64). PGD_2_ binds to smooth muscle cells leading to vasodilatation and bronchoconstriction and can also bind the chemoattractant receptor-homologous molecule expressed on TH2 lymphocytes (CRTH2) inducing Th2 cytokine production and further promoting activation of MCs and PGD_2_ production in asthma (64).

## PGD_2_ Receptor 2 (CRTH2) Antagonists in Severe Asthma: A Novel Therapeutic Approach

A recent novel class of drug that is undergoing phase II studies in adults and seems attractive for pediatric severe asthma is the CRTH2 antagonists (71). CRTH2 is present on MCs, but also on eosinophils, Th2 cells, and ILC2. Given the overwhelming evidence that pediatric severe asthma is associated with severe atopy, is eosinophilic, and associated with significantly increased numbers airway of ILC2, the strategy of blocking the CRTH2 receptor is very appealing. A randomized, parallel double blind placebo-controlled trial of a CRTH2 antagonist in adults with persistent, moderate-to-severe asthma, and an elevated sputum eosinophil count showed a reduction in sputum eosinophils in the active group, and no associated significant adverse effects (72). Another potential advantage of these compounds is that they can be administered orally. However, clinical efficacy is yet to be proven. Small phase II trials have suggested efficacy in achieving an improvement in symptoms and lung function (73), but they did not target the population that had eosinophilic or type 2 high diseases. It is increasingly apparent that as more and more add-on therapies become available and may potentially be utilized in children, the need to identify the right drug for the right patient phenotype will be essential (74).

## Regulatory T Cells

After TCR engagement, activation of T cells can be suppressed by regulatory CD4+CD25+ T cells (T_reg_) (75). Therefore, T_reg_ cells can control allergen-specific immune responses and low numbers or dysfunctional T_reg_ cells may contribute to allergic disease and asthma. However, few studies have investigated the role of T_reg_ cells in pediatric severe asthma. Low T_reg_ cells in blood and sputum as well as impaired suppressive function during exacerbations have been reported in severe refractory asthmatic adults compared to healthy controls (76). In contrast, another small study comparing numbers of T_reg_ cells in BAL from moderate to severe asthmatic adults compared to mild asthmatics reported that T_reg_ cells were increased in the severe group (77). In asthmatic children, T_reg_ cells were lower in BAL (78) and blood (79). Peripheral T_reg_ cell levels were lower compared to healthy controls, especially in the acute phase and in the severe group. Th1/Th2 ratio correlated positively with T_reg_ cells and negatively with disease severity (79).

T_reg_ cells can act through perforin-mediated cytolysis as well as IL-10 and TGF-β. IL-10 is a potent anti-inflammatory cytokine expressed by several cell types, including T cell subpopulations. IL-10 suppresses the production of inflammatory cytokines, the DC-mediated antigen presentation to T cells as well as the function of MCs and eosinophils (80). In addition, IL-10 inhibits IgE and favors IgG4 to IgE (81). Lower levels of IL-10 are produced by macrophages and mononuclear cells from asthmatics (82, 83). Defective IL-10 expression has been associated with increased steroid resistance in children with severe asthma (84), and vitamin D enhances the frequency of both IL-10+ and Foxp3+ T_reg_ cells in children with severe asthma (85). In a translational setting, these data suggest that vitamin D supplementation may be effective in enhancing the frequency of T_reg_ cells in pediatric severe asthma.

Other studies have suggested that TGF-β, rather than IL-10, may be more important and serve as a biomarker of asthma control in atopic asthma (86). TGF-β is a pleiotropic cytokine with numerous functions that are vital in maintenance of pulmonary homeostasis, such as inhibiting Th2 and Th1 cell responses or inhibiting IgE production (87). In children, polymorphisms in TGF-β2 have been associated with atopic asthma (88). PCR analysis of bronchial and nasal epithelial cells concluded that TGF-β2 was differentially expressed in pediatric asthmatics compared to atopic non-asthmatics and healthy children (89). However, a much better understanding of the complex TGF-β signaling network in pediatric severe asthma is required before specific molecules can be targeted in a valid clinical study.

## Airway Remodeling: Mechanisms and Therapeutic Targets

Children with severe asthma have evidence of all of the structural airway wall changes (remodeling) that are apparent in adults. They have increased thickness of the reticular basement membrane (RBM) (8, 90), increased ASM (8, 91, 92), goblet cell and submucosal gland hyperplasia (93), and evidence of angiogenesis (67). Of these changes, increased bronchial ASM has been closely related to worse lung function and greater bronchodilator reversibility (91, 92, 94).

### Relationships between Inflammation and Remodeling

It is often proposed that remodeling occurs as a consequence of chronic airway inflammation. Payne et al. (90) compared RBM thickness in 19 children with difficult asthma prescribed high-dose inhaled steroids (6–16 years) and 10 age-matched non-asthmatics children with healthy, steroid-naive asthmatic adults, and life-threatening asthmatic adults. RBM thickness was not associated with severity, asthma symptoms, age, or airway inflammation. Fedorov et al. (95) compared bronchial biopsies between non-asthmatic, moderate, and severe asthmatic children (5–15 years) and showed excess deposition of interstitial collagen in the RBM occurred early in life but did not correlate with submucosal eosinophils and suggested that RBM thickness is established early in life due to an abnormal EMTU. Both studies proposed that remodeling is dissociated from eosinophilic inflammation. The dissociation between airway remodeling and eosinophilic inflammation has been demonstrated in a mouse model in which HDM-induced airways remodeling was equivocal in eosinophil-deficient and wild-type mice (96). Mechanistic data from a neonatal mouse model of inhaled HDM exposure have shown that remodeling is unlikely a consequence of inflammation, but that both processes occur in parallel (97). Remodeling can, therefore, develop in the absence of an inflamed airway with just excessive bronchoconstriction (98), and thus, there is an urgent need for therapies that can target structural changes alone, as many children with severe asthma remain symptomatic with significant airway hyperresponsiveness in the absence of inflammation (99).

### Airway Smooth Muscle

The importance of targeting ASM remodeling as a therapeutic approach is made apparent by the very consistent association with increased ASM and worse lung function in both adult and pediatric studies. Increasing ASM has also been associated with lower serum vitamin D levels and worse asthma control in children (94). Mechanistically, a relationship between increased airway remodeling and a vitamin D-deficient diet has also been shown in a neonatal mouse model of HDM-induced allergic airways disease (100). These data suggest studies that focus on investigating ASM function in pediatric severe asthma are likely to be helpful in discovering novel therapeutic targets. In addition, that vitamin D supplementation to achieve normal serum levels in children with severe asthma is an important consideration as it may minimize remodeling.

There is evidence that ASM function is specifically impaired in adult severe asthma, and that the mechanism is related to glucocorticoid resistance, whereby glucocorticoid receptor expression is reduced with impaired nuclear translocation (101). In contrast to ASM, few functional consequences have been reported in association with the thickness of the subepithelial RBM. Moreover, increased thickness is not an isolated finding in asthma, although the degree of thickening is greater in severe asthma, this feature may also be present in children with cystic fibrosis (102) and adults with COPD (103). Thus, it is difficult to know the impact that therapies, which target increased RBM thickness may have on disease manifestation.

## Importance of Allergy in Pediatric Severe Asthma: Mechanisms and Anti-IgE Antibody Therapy

More than 85% of children with severe asthma are atopic, defined by serum IgE antibodies and a positive skin prick test to common aeroallergens (8). One of the key clinical features that allows distinction between children with difficult asthma (poor control with poor adherence) and severe therapy resistant asthma (poor control despite good adherence) is significantly more severe asthmatics were polysensitised to several allergens, and more patients had food allergy (104). However, perhaps the most important distinctive feature of severe asthma is when atopy is quantified, rather than assessed as simply being present or not (105, 106). Children with severe asthma have a much worse and higher allergic burden (107). This suggests allergic sensitization plays a critical role in the pathogenesis of severe asthma in children (108). The role of allergy in pediatric severe asthma needs to be understood to help identify underlying mechanisms of disease progression, which will impact both on the choice of add-on therapies for these patients, but also on the discovery of novel therapeutics. In this regard, the one therapy that has been approved for use in children with severe asthma is the recombinant DNA-derived humanized monoclonal antibody against IgE (omalizumab), which works by reducing the quantity of cell-bound IgE, downregulation of high-affinity IgE receptors FcεRI on MCs, basophils, and DCs, and prevention of mediator release from effector cells (109, 110). Decreased sputum and bronchial eosinophils, as well as T cells were observed in adult bronchial biopsies after omalizumab (109). In a study (111) involving 334 children aged 6–12 years with moderate-to-severe atopic asthma treated with beclomethasone dipropionate and omalizumab or placebo, omalizumab reduced number of exacerbations as well as the frequency of exacerbations when withdrawing ICS. Another smaller 16-week study in children with severe asthma reported that omalizumab allowed a significant reduction in daily prednisolone dose and improved control and life quality (112).

However, the current licensed indication requires serum IgE levels to be within a set range, the maximum being 1,500 IU/ml. At least one-third of children with severe asthma have an IgE greater than 1,500 IU/ml because of severe and multiple allergies (8). In addition, approximately one-third of children who are eligible and are given a trial of treatment do not have a clinical response (113, 114). Therefore, there is still a subgroup of children with very severe disease and marked morbidity for whom currently no licensed add-on therapies are available. Interestingly, given the burden of monthly, or two weekly injections posed by omalizumab, and that specific subgroups, adolescents in particular, who are at high risk of asthma death, but more likely to be non-compliant with maintenance therapy, an approach of giving omalizumab prior to the Autumn increase in asthma exacerbations has been efficacious (115). Omalizumab therapy was associated with improved IFN-α responses to rhinovirus. However, changes in allergen-stimulated cytokine responses in peripheral blood T cells, or changes in T regulatory cells were not seen (116). Given the clinical benefit, this suggests the effects of omalizumab are unlikely *via* an impact on T cell responses, and more likely *via* other immune effector cell types such as MCs (116).

## Allergy in Preschool Wheeze: A Possible Window for Asthma Prevention?

Preschool children with wheezing disorders may or may not progress to develop asthma. Airway inflammation can be assessed in BAL and endobronchial biopsies from children with severe wheezing. BAL from wheezing children contains increased lymphocytes, polymorphonuclear cells, and macrophages/monocytes as well as LT B4, C4, PG E2, and the potentially epithelial-derived 15-hydroxyeicosatetraenoic acid were all increased (117). Bronchial biopsy studies in infants under 2 years with severe wheeze have reported an absence of RBM thickening and eosinophilic inflammation (118). However, when older children at a median age of 3 years with severe recurrent wheezing were compared to non-wheezing controls, they had increased airway eosinophils and RBM thickness (119). Birth cohort studies have repeatedly shown that the most prominent risk factor for progression of preschool wheeze to asthma is early allergen sensitization (120). Also, in a similar manner to older children with severe asthma, the risk of developing asthma and the greatest reduction in lung function is in those preschool wheezers who have both early and multiple allergic sensitization (121, 122). Unfortunately, targeting eosinophils with early inhaled steroids is not disease modifying (123), and this is explained by the mechanistic data that shows an absence of eosinophils does not impact the phenotype of allergic airways disease (96). However, given the definite association between early allergic sensitization and progression of preschool wheeze to asthma, several alternative interventions have been proposed to achieve disease prevention (124). One of these, to investigate the role of omalizumab in preschool wheeze to achieve disease modification is currently being tested in a clinical trial (125).

## Emerging Therapies for Severe Asthma

According to the ATS/ERS guidelines, severe asthmatics have poor control despite treatment with high-dose inhaled or oral corticosteroids combined with LABAs. A summary of the emerging add-on therapies that are being trialled for severe asthma is provided below. However, it is important to remember that, of these, only omalizumab is currently licensed for use in children with severe asthma.

### Muscarinic Antagonists

These drugs act as bronchodilators by non-specifically antagonizing the muscarinic acetylcholine receptor and inhibiting smooth muscle cell contraction and mucus secretion. Short-acting muscarinic antagonists (SAMAs), such as ipratropium bromide, can be used in severe asthmatic children and adults during asthma exacerbations (126) and to reduce β-agonist doses in order to avoid side effects, but they are less effective than inhaled beta-agonists (127). In 1998, Qureshi et al. already reported that adding this drug to a combined therapy of albuterol and corticosteroids decreased hospitalizations for severe asthmatic children (aged 2–18 years). More recently, a meta-analysis reported that the combination of SAMAs and SABAs in children during exacerbation improves lung function and reduces the risk of tremor and the risk of admission (128).

Longer-acting muscarinic antagonists are an interesting option as controller medications. Inhaled Tiotropium (Spiriva Respimat^®^) was first indicated in COPD treatment. Currently, it is approved as an add-on maintenance bronchodilator in adults with asthma taking ICS/LABAs and who have experienced at least one severe exacerbation in the previous year. It was shown to improve lung function and symptoms in uncontrolled moderate-to-severe asthmatic adults and reduced the risk of exacerbations in those treated with ICS/LABAs (129, 130). US FDA has recently approved it as an asthma maintenance treatment in children aged 6 years and over (131). However, it has not been approved in Europe. Its efficacy as an add-on therapeutic in pediatric severe asthma remains unknown. Pediatric trials that will allow selection of patients that are most likely to benefit are needed.

### Immunomodulators

Molecular-based therapies, which allow treatment according to the predominant inflammatory phenotype, are of particular interest in severe asthmatic children who do not respond to standard therapy. Apart from specificity, they provide long-term control and allow a reduction in ICS and oral steroid dose. An important aim in the utility of novel therapeutics for children is not just as add-on treatments that will allow disease control, but also as steroid sparing therapies to minimize the significant adverse effects of high-dose corticosteroids. Several antibody-based treatments are available.

#### Neutralizing IgE: Omalizumab

The mechanism of action and specific utility of omalizumab in pediatric severe asthma has been discussed in the section on allergy in children above.

In the UK, omalizumab is indicated as an add-on therapy to improve control in adults and children aged 6 years and over with severe persistent confirmed IgE-mediated asthma, and who need continuous or frequent treatment with oral corticosteroids (defined as four or more courses in the previous year) (132). The predominant benefit is a reduction in exacerbations. However, limitations for children include the upper limit of serum IgE for which it can be prescribed and at least one-third do not have a clinical response.

#### Blocking IL-5 Signaling: Mepolizumab

Mepolizumab is an anti-IL-5 humanized monoclonal antibody that reduces circulating eosinophils. It is indicated as an add-on to standard therapy in severe refractory eosinophilic asthma in adults when the blood eosinophil count is ≥300 cells/μl in the previous 12 months and ≥4 exacerbations needing systemic corticosteroids in the previous 12 months need for continuous oral corticosteroids equivalent of more of prednisolone 5 mg/day over the previous 6 months (133). Exacerbations in adults decreased without improvement in FEV_1_ nor quality of life. The data for efficacy in children are currently lacking, and clinical trials that address this unmet need are urgently needed.

#### Blocking Th2 (IL-13 and IL-4) Signaling: Lebrikizumab, Dupilumab, and Pitrakinra

Lebrikrizumab, a humanized monoclonal antibody against IL-13, has been evaluated for severe asthmatic adults. Initial studies showed Lebrikizumab treatment resulted in improvement in lung function in adults with uncontrolled asthma taking ICS, especially in those with higher levels of serum POSTN. There was also a decrease in exacerbation rates in those patients with higher blood eosinophils and IgE levels (134). However, subsequent confirmatory studies in adults have been disappointing (135) and it is not yet indicated for adult severe asthma.

Animal studies have shown that IL-4 can induce IL-13-independent AHR and goblet cell hyperplasia, suggesting dual inhibition of both IL-4 and IL-13 could suppress these events (136). Dupilumab is a human monoclonal antibody against the IL-4 receptor α chain (IL-4Rα) blocking downstream signaling *via* both the IL-4 and IL-13 receptors. In 2013, Wenzel and colleagues performed a 12-week study (phase 2a) to asses Dupilumab in persistent moderate-to-severe asthmatic adults with high blood (≥300/μl) or sputum (≥3%) eosinophils (Th2 high disease) (137). Dupilumab was effective in reducing exacerbations, increasing lung function and reduced Th2-associated inflammation, as well as allowing a reduction and/or stopping of maintenance dose of ICS while maintaining improvement of asthma. More recently, in a 24-week study (phase 2b) in uncontrolled persistent asthmatic adults, injection with Dupilumab every 2 weeks as an add-on therapy to medium-to-high-dose ICS and LABAs led to improvement of FEV1, reduction in exacerbations, and better asthma control regardless of baseline eosinophil count (138). During the treatment patients with at least 300 eosinophils/μl at baseline had an increase in blood eosinophils, so the next clinical trial (139) excluded patients with high eosinophils and also included children (from 12 years). Efficacy of this antibody in uncontrolled persistent asthmatic children (aged from 6 to 12 years) will be assessed in a clinical trial starting on 2017 (139). Pitrakinra is a recombinant human IL-4 antibody containing mutations that allow it to prevent the assembly of IL-4Rα with either IL-2Rγ or IL-13Rα (140). In a recent study, a dose–response for asthma exacerbations was identified in a specific subgroup according to the SNPs genotype in *IL4RA* gene (141).

#### Blocking Th17 Signaling: The Role of IL-17 and Neutrophils in Pediatric Severe Asthma

In adults with severe asthma, high levels of IL-17A have been reported in sputum, BAL fluid, and peripheral blood (142–144), and this was associated with increased disease severity (145). In children, IL-17A has been reported to induce neutrophilic airway inflammation and promote steroid resistance (146). Levels of IL-17A in sputum, nasal wash, and plasma as well as levels of circulatory T cells expressing IL-17 were studied in children with moderate asthma, and it was suggested that IL-17 could be associated with asthma severity (147). In contrast to the data from studies in adults, we have shown levels of IL-17A are not elevated in either the BAL or endobronchial biopsies of children with severe asthma compared to non-asthmatic controls (148). However, we did show increased expression of IL-17Rα in the airway submucosa and epithelium of children with severe asthma. Furthermore, numbers of neutrophils were also similar in the BAL and submucosa of children with severe asthma and controls (8, 148). However, there was a subgroup of patients with increased numbers of neutrophils only within the epithelium, and these patients had better symptom control improved lung function, symptom control, and were prescribed lower dose maintenance inhaled steroids. Therefore, unlike adult severe asthma, neutrophils might be beneficial in pediatric severe asthma pathophysiology. Therefore, the role of neutrophils and IL-17 is not completely understood in children with STRA and requires further investigation before therapies that are either antineutrophilic or block IL-17 are tested. Interestingly, Brodalumab, a human anti-IL-17 receptor monoclonal antibody, has shown no benefit in adults with moderate-to-severe disease (149).

#### Anti-TSLP Antibody: AMG157

The monoclonal antibody AMG157 blocks the binding of TSLP with its receptor and has been shown to reduce bronchoconstriction and attenuate the early and late phase response in allergic asthmatic adults (150). It also reduced blood and sputum eosinophil counts and FeNO before allergen challenge, suggesting that TSLP may be an important upstream regulator of type 2 inflammation in the airways. The efficacy of AMG157 is currently being assessed in adolescents (12–17 years old) with mild-to-moderate asthma (151) and will be assessed in uncontrolled severe asthmatic adults (152). The efficacy of blocking TSLP in children with severe asthma remains to be seen. There is no evidence to date that levels of TSLP are elevated in the airways of children; therefore, a direct extrapolation of findings from adult studies to pediatric studies may not be beneficial.

#### Anti-PGD_2_

Prostaglandin D2 is a lipid inflammatory mediator produced by cyclooxygenases and PGD_2_ synthases mainly in MCs, but also in Th2 cells, macrophages, and much less in eosinophils and basophils. They subsequently bind to D prostanoid 1 and chemoattractant receptor-homologous molecule expressed on Th2 cells (CRTH2) receptors triggering anti-inflammatory effects as well as pro-inflammatory effects through PGD_2_-CRTH2 pathway, which is upregulated in asthma (153). This is a particularly attractive therapeutic for children and has been discussed in detail above in the section on airway inflammation in pediatric severe asthma.

## Future Directions

We have discussed two add-on treatments that are currently available for use in adults that mechanistically are attractive therapeutic options for children with severe asthma and need to be pursued. Monoclonal antibodies to IL-5 or its receptor, and CRTH2 antagonists, both of which may help to ameliorate the persistent steroid resistant eosinophilia and elevated Th2 and ILC2 cells that are apparent. Although both types of interventions should be efficacious, we know that omalizumab, the currently licensed add-on treatment for children, does not work or cannot be used in about 50%, so similarly, it is likely that the other two therapeutics will also only work in a subgroup. In contrast to adult studies, given the current mechanistic data from children, it seems much less likely that antibodies that target IL-13, IL-4, or IL-17 will be beneficial in children. This is because these data showing elevated levels of these mediators in pediatric airways are scarce. A molecular target for which therapeutic agents are not currently available, but would certainly be worth pursuing for children, is IL-33. This is because it is elevated in children, is relatively steroid resistant, promotes airway remodeling (56), and is more specifically associated with the sub-phenotype of severe asthma with fungal sensitization (57).

It is essential to remember that pediatric severe asthma is markedly heterogeneous, and in contrast to adult disease, our current understanding of the underlying sub-phenotypes and endotypes is very limited. This is because it is a rare disease, but also because mechanistic studies that use either age appropriate experimental models or airway samples from children are a challenge to undertake. We know that there are distinctions between the factors that drive pediatric and adult disease, most importantly, pediatric disease is present in the context of a maturing immune system and during lung growth and development. Given the acknowledged heterogeneity and the relatively small number of patients that are affected, it is essential that we now undertake multicenter, national and if possible, international unified studies to assess the efficacy of novel therapeutics and to investigate the mechanisms of action of these drugs in children. Only such an approach will allow us to understand the mechanisms mediating disease, and to identify important endotypes, which will allow us to stratify patients and ensure add-on treatments are accurately targeted to achieve effective personalized medicine.

## Author Contributions

AM and SS contributed equally to this work.

## Conflict of Interest Statement

The authors declare that the research was conducted in the absence of any commercial or financial relationships that could be construed as a potential conflict of interest.
